# Public awareness and support for environmental protection—A focus on air pollution in peninsular Malaysia

**DOI:** 10.1371/journal.pone.0212206

**Published:** 2019-03-14

**Authors:** Yunn Shin Jocelyne Chin, Laura De Pretto, Vivek Thuppil, Matthew J. Ashfold

**Affiliations:** 1 School of Environmental and Geographical Sciences, University of Nottingham Malaysia, Semenyih, Selangor, Malaysia; 2 Department of Applied Psychology, University of Nottingham Malaysia, Semenyih, Selangor, Malaysia; 3 Mindset Interdisciplinary Centre for Environmental Studies, University of Nottingham Malaysia, Semenyih, Selangor, Malaysia; 4 Department of Psychology, School of Social and Health Sciences, Leeds Trinity University, Leeds, United Kingdom; 5 School of Psychology, University of Nottingham Malaysia, Semenyih, Selangor, Malaysia; University of Pittsburgh, UNITED STATES

## Abstract

As in many nations, air pollution linked to rapid industrialization is a public health and environmental concern in Malaysia, especially in cities. Understanding awareness of air pollution and support for environmental protection from the general public is essential for informing governmental approaches to dealing with this problem. This study presents a cross-sectional survey conducted in the Klang Valley and Iskandar conurbations to examine urban Malaysians’ perception, awareness and opinions of air pollution. The survey was conducted in two languages, English and Malay, and administered through the online survey research software, Qualtrics. The survey consisted of three sections, where we collected sociodemographic information, information on the public perception of air quality and the causes of air pollution, information on public awareness of air pollution and its related impacts, and information on attitudes towards environmental protection. Of 214 respondents, over 60% were positive towards the air quality at both study sites despite the presence of harmful levels of air pollution. The air in the Klang Valley was perceived to be slightly more polluted and causing greater health issues. Overall, the majority of respondents were aware that motor vehicles represent the primary pollution source, yet private transport was still the preferred choice of transportation mode. A generally positive approach towards environmental protection emerged from the data. However, participants showed stronger agreement with protection actions that do not involve individual effort. Nonetheless, we found that certain segments of the sample (people owning more than three vehicles per household and those with relatives who suffered from respiratory diseases) were significantly more willing to personally pay for environmental protection compared to others. Implications point to the need for actions for spreading awareness of air pollution to the overall population, especially with regards to its health risks, as well as strategies for increasing the perception of behavioural control, especially with regards to motor vehicles’ usage.

## Introduction

Over the last half-century, many countries have transformed from an agrarian-based rural economy towards an industrial-based urban economy [1]. As a consequence, various human activities in these countries now emit harmful particulates (often defined as PM_10_ or PM_2.5_) and gases (e.g. ozone, nitrogen dioxide) and thus severely pollute the air [2]. The World Health Organization (WHO) noted that each year poor air quality causes over seven million premature deaths globally, with higher impacts in developing nations [3], such as in Indonesia [4], India [5] and China [6]. The detrimental impacts on human health strongly correlates with the polluted environment, which subsequently degrades life satisfaction [7].

As with other developing nations, Malaysia has experienced rapid industrial development and urbanization, and aims to become a developed country by the year 2020 [8]. This economically beneficial development process, however, has also polluted the atmosphere (see [9]), especially in conurbations (e.g. [10]), of which the largest three are the Klang Valley (containing Kuala Lumpur and adjoining cities), Greater Penang, and Iskandar Malaysia (containing Johor Bahru and adjoining cities). These three urban agglomerations contain about a third of the overall country’s population. While comprehensive data on pollutant emissions are limited, mobile sources have been identified as the main contributor (70–75%) to urban air pollution [11, 12].

Malaysia also experiences regional air pollution, such as severe ‘haze’ episodes mostly caused by widespread forest fires in Indonesia (e.g. [13]). The 1997 Southeast Asian haze event was the first such event [9]. Subsequent regional haze events reoccurred, with the most recent one in 2015 being notably the worst since 1997 [14]. In addition to releasing harmful pollutants (e.g. [15]; [16]), for about two weeks of the two-month period of September–October 2015, the fires producing the haze were also emitting up to 20 million tonnes of carbon dioxide-equivalent per day, surpassing the entire output of the United States for that period [17]. These environmental effects, combined with socioeconomic and health impacts, have caused international political concern among the member nations of the Association of Southeast Asian Nations (ASEAN) over the haze [18, 19].

Official statistics for 2013 list respiratory illnesses as the second highest principal cause of both hospitalization (12.4% of cases) and mortality (21.7%) in Malaysia [20]. There is also growing evidence for the impacts of air pollution in Malaysia on health related to respiratory organs (e.g. [21]). Furthermore, during haze episodes, a positive correlation has been observed between pollution levels and increases of asthma, acute respiratory infection, and conjunctivitis outpatient visits in several states [11]. Other lines of evidence show Malaysian commuters exposed to the haze reported substantially greater adverse health experiences, with complaints such as headache, coughing, and breathing difficulties [22]. The 2015 Southeast Asian haze event had particularly serious health impacts, with smoke exposure during the episode estimated to have resulted in an excess of 6,500 deaths in Malaysia [23], substantially higher than the 2,300 deaths estimated from the 2006 Southeast Asian haze event [23].

Pollution is controlled in Malaysia through various environmental policies and laws, such as the Environmental Quality Act 1974, with subsidiary legislation such as the Malaysian Ambient Air Quality Standard 2013, Environmental Quality (Clean Air) Regulations 2014, and the like. These regulatory approaches act to mitigate and supervise pollutants (including particulates, ozone and nitrogen dioxide) emitted from different sectors [24]. Despite this regulatory framework, Malaysia still suffers from air pollution. As suggested by Inglehart [25], the success of government efforts and policies designed to resolve environmental issues can only be achieved with citizens’ support for environmental protection. It follows that government efforts to enhance air quality in Malaysia will be helped by an improved understanding of the awareness and support for environmental protection among the country’s citizens. Studies conducted in Malaysia on air pollution have mainly focused on the environmental and atmospheric aspects of pollution, particularly with a focus on quantifying the level and nature of pollutants [[9], [10], rather than the social aspect of pollution investigating topics such as citizens’ attitudes.

The Theory of Planned Behaviour in psychology [26, 27, 28] explains that people’s positive attitudes will lead to good behavioural intentions [29, 30]. When considering economic valuation, the environmental attitudes-behaviour link explained in the Theory of Planned Behaviour translates into willingness to pay (WTP) for environmental protection when pro-environmental attitudes are present [31, 32]. In various studies, this WTP for environmental protection has been linked to affluence [33, 34, 35]. Awareness of environmental problems has been shown to be positively correlated with factors such as age, education level, health conditions and parenthood in various studies conducted in developing countries such as Indonesia [36], Turkey [37], Malaysia [38] and China [39].

Studies of public perception, awareness, and attitudes (including WTP) towards air pollution are rare in Malaysia. Thus, this study attempts to explore this topic by understanding the public’s perception of current air pollution, their environmental awareness, and attitudes towards environmental protection. The rationale for conducting this study is that the implementation of any programme or legislation could not be successful without public awareness and support for environmental protection. In short, only citizens who are well aware of the situation and fully dedicated to their right to a quality environment could then drive possible environmental protection practices. More detailed insights gained from this type of study can help to frame and design the most appropriate policy options.

## Materials and methods

### Participants

Malaysians from the Klang Valley and Iskandar Malaysia conurbations were targeted as survey subjects because, as residents of among the largest urban and industrial areas in Malaysia, they are likely to be most affected by air pollution from vehicular and industrial emissions. The Klang Valley (KV hereafter), as the metropolitan area of Kuala Lumpur is usually referred to, is near the west coast of Peninsular Malaysia and comprises the federal territories of Kuala Lumpur and Putrajaya, and the highly urbanized towns and cities in Selangor state surrounding these federal territories. Iskandar Malaysia (IM hereafter), occupies the southern tip of Peninsular Malaysia facing the island nation of Singapore, and includes the city of Johor Bahru and surrounding municipal areas. Malaysian citizens residing within these survey sites were eligible to participate in the study. The sample includes Malaysian participants from the three major ethnicities, i.e. Malay, Chinese and Indian, present in the survey areas. Non-Malaysian residents were excluded from the study, based on considerations related to methods and implications of the study. Methodologically, targeting Malaysians only helps making the sample more uniform, which means other related variables are controlled. For instance, non-Malaysian individuals could have moved to Malaysia too recently (or plan to stay for a too short amount of time) to be really affected by the air pollution. Or they could have developed their attitudes towards air pollution based on their experience in a country or context where they lived prior to moving to Malaysia. In terms of implications of the study, knowing specifically what Malaysian citizens think is more relevant for policy makers. Malaysians are the group of people being affected and controlled by the government’s regulations, plans and incentives, thus targeting Malaysians only provides the authors with a more applied view on attitudes towards environmental protection, especially with regards to factors related to taxes (as measured by some of the items in the survey).

### Instrumentation

The framework of the survey instrument was adapted from our recent study [38] that was carried out in Malaysia with similar objectives. This survey was created via an online research software, Qualtrics (Qualtrics LLC, Provo, UT, USA) in the form of a self-administered questionnaire. Participants were expected to complete the survey without any guidance provided by the researchers. The online questionnaire was designed in two languages: English and Malay, in order to ensure that targeted respondents were able to comfortably answer the questions with their preferred language.

The survey questionnaire (S1 and S2 Files) contained three sections. Section A, on *Background and demographic descriptors*, was composed of 13 questions to obtain sociodemographic information from the respondents. Section B was in two parts. Section (B.1), on *Air pollution perception*, was composed of 2 questions designed to understand public perceptions on the condition of air quality and the factors causing air pollution in each study site. Section (B.2), *on Air pollution awareness*, was composed of 9 true/false/no-answer questions to examine public awareness of air pollution and its associations with health, economic costs and governmental pollution management practices. Section C was an *Environmental protection attitude questionnaire* composed of 18 statements on a 5-point Likert scale, including statements created by the researcher and statements adapted from the International Social Survey Programme (http://www.issp.org) 1993 & 2000, World Values Survey (http://www.worldvaluessurvey.org) Wave 2, 4, 5 & 6, and De Pretto *et al*. [38]. As suggested by Maloney *et al*. [40], a scale aiming to investigate attitude should comprise three components–conative, affective and cognitive. Here, the cognitive component consists of 8 statements while affective and conative consist of 5 each, adding up to 18. The intention was to examine general support for environmental protection and willingness-to-pay (WTP) towards environmental protection pertinent to air pollution.

### Data collection

The survey was performed for a total of 80 days between November 2016 and January 2017. Prior to the commencement of actual data collection, two rounds of pilot studies were conducted with 20 people. This was to ascertain that all questions could be correctly interpreted and rationally answered. The survey was then distributed via Qualtrics with the use of an anonymous link through social media platforms and personal networks. In total, 327 responses were collected. Each survey took an average of 15 minutes to complete. This research was reviewed and approved by the Science and Engineering Research and Ethics Committee, University of Nottingham Malaysia Campus.

### Data treatment and analysis

Data was first managed by grouping and cross-checking the recorded responses in Qualtrics. Responses with extensive missing answers (a total of 28) or with all identical answers (a total of 43), were considered as unqualified and removed from further analysis. Questionnaires filled out by respondents who are not residing in KV or IM (a total of 38) were also deemed ineligible. As a screening measure before formally starting the survey potential participants were asked to continue only if they were Malaysian. There was no question pertaining nationality in the survey itself. However, the screening proved not to be error free, as four participants, in answering a question about their ethnicity, declared their (non-Malaysian) nationality instead (confusing “nationality” and “ethnicity”). This information was used to discard these four participants from the study. So in total, 113 questionnaires were discarded. This provided a final sample (S3 File) size of 214, with 97 surveys collected from KV (45% of the total respondents) and 117 surveys collected from IM (55%). Data analyses were done using the Statistical Package for the Social Sciences (SPSS) version 23 (IBM Corp, Armonk, NY).

In Section B.1, respondents were required to rank the significance of four factors based on what they think contributes most to the air pollution at their respective place of residence. Their responses were then normalized using the ‘Relative Priority Index’ (RPI) method, which is adapted from De Pretto *et al*. [38]. With regards to the awareness scale in Section B.2, a score of 1 was awarded for each right answer, -0.2 for wrong answer and zero for any ‘no-answer’ option chosen. This mild negative scoring system was used to distinguish participants who did not know the answer and those who made an error. An average ‘awareness’ score was then calculated for every respondent. This approach has been adopted in various past research [38 39, 41].

In Section C, scores on various statements related to environmental protection attitudes were determined from the 5-point Likert scale answers. A higher score indicates a more positive attitude. Statements 2, 5, 7, 11, 12, 14 and 18, which refer to negative attitudes towards environmental protection, were reverse scored. We initially intended to develop a scale for measuring the level of support for environmental protection. In exploring this possibility we used Cronbach’s alpha, which is “perhaps the most widely used measure of the reliability of a scale” ([42], p. 99). A lack of homogeneity in the items that comprise a scale cause reliability errors. It is assumed that the items on a scale are a random sample of all possible items that could measure a certain attribute; thus, those items should be highly correlated. While judgment on an ideal value of alpha needs to be made on a case to case basis, it is commonly understood in the social sciences that good alpha values should be between .7 and .9. However, in the current study the pool of items as a whole (after the removal of outliers) had a low internal consistency (α = .494), which would not significantly increase with the deletion of a few items. For this reason, results will be mainly presented based on descriptive (rather than inferential) statistics.

Nonetheless, 5 out of the 18 items, which were meant to represent the conative component of the attitude scale (denoting behavioural intentions), possessed adequate reliability (α = .62). This level of alpha, which is greater than .6, is acceptable for measures used for the first time in a new culture [43]. Our conative component is composed of items referring to the willingness to pay (WTP) for environmental protection. Two of the items were derived from the World Value Survey (WVS), Wave 4 and Wave 5, (2000–2004; 2005–2009), two from the International Social Survey Programme (ISSP, 1993), and one was self-developed. The items derived from WVS and ISSP were slightly modified in order to adapt them to the Malaysian socio-cultural context. They were administered for the first time to a Malaysian sample as a cluster. Items included “I do not mind paying more money to use better quality gasoline which leads to less pollution” and “I am willing to accept cuts in my standard of living in order to protect the environment” (see Table 1 for the complete list of items). In addition to being used in a new culture for the first time, the scale is composed of only five items. Cortina [44] (as cited in [45]), stated that a low number of items in a scale will lead to a low Cronbach’s alpha value. We believe that a Cronbach’s alpha of .62 is acceptable for a scale composed of five items, using our judgment consistently with the recommendations provided by Streiner [42]. Thus, the five items composing WTP were treated as a scale, and, with regards to this scale, inferential statistics was used in addition to descriptive statistics to produce more insightful results.

**Table 1:** List of items related to attitude towards environmental protection.

Item n°	Item		Source of item
1.	Taking care of the environment is something I really care about.		Self-developed.
2.	In order to protect the environment Malaysia needs economic growth.	(R)	ISSP 1993, V13.
3.	I would contribute part of my income if I were certain that the money would be used to prevent atmospheric pollution.	(C)	WVS Wave 5: 2005–2009, V105.
4.	The air quality in Malaysia is getting better because of modern science and technology.		ISSP 1993 and ISSP 2000.
5.	Malaysians worry too much about industrial development polluting the atmosphere and degrading humans’ health.	(R)	ISSP 2000.
6.	Educating younger generations about the knowledge of environmental protection (e.g. encourage carpool) is important.		Self-developed.
7.	Nothing can be done by me or my family/friends to improve the current atmospheric situation.	(R)	De Pretto et al. (2015).
8.	I do not mind an increase in taxes if the extra money is used to prevent further atmospheric pollution.	(C)	WVS Wave 4: 2000–2004, V34; ISSP 1993 and ISSP 2000.
9.	Protecting the environment should be given priority, even if it causes slower economic growth and some loss of jobs.		WVS Wave 6: 2010–2014, V81.
10.	I often cut back on driving a car for environmental reasons.	(C)	ISSP 1993, V59.
11.	There is no point in doing what I can for the environment unless everyone does the same.	(R)	ISSP 1993, V27.
12.	Haze is a fair price to pay for economic development.	(R)	De Pretto et al. (2015).
13.	I do not mind paying more money to use better quality gasoline which leads to less pollution.	(C)	Self-developed.
14.	The economic growth of Malaysia is currently more important than environmental protection.	(R)	Self-developed.
15.	I am willing to accept cuts in my standards of living in order to protect the environment.	(C)	ISSP 1993, V26.
16.	Air pollution caused by cars is extremely dangerous for health.		Self-developed.
17.	I have confidence that the air quality in Malaysia will improve before Wawasan 2020.		Self-developed.
18.	Malaysia government has to reduce atmospheric pollution but it should not cost me any money.	(R)	WVS Wave 2: 1990–1994, V14.

Note: Reversely scored items are indicated with (R); Items part of the WTP scale are indicated with (C); ISSP = International Social Survey Programme, WVS = World Values Survey; items derived from WVS and ISSP were slightly modified in order to adapt them to the Malaysian socio-cultural context.

The associations of all demographic factors (the independent variables) on awareness and attitude scores were analysed using a univariate analysis in the form of t-tests, chi-squared tests of association or ANOVA tests.

## Results

### Characteristics of respondents

The sample characteristics are summarized in Table 2. Most of the respondents were between 21–55 years old (68.2%), equipped with a tertiary educational attainment (65.4%), and worked in non-environmental private industry (55.7%). The employment status of the entire sample was primarily student (46.3%) or full-time worker (30.8%). In terms of household monthly income, over 50% of the sample reported an amount of ≤ Malaysian Ringgit 5000 per month. Household vehicle ownership was high among the entire sample (95.6%), with 80.7% of vehicles using petrol as fuel. The proportion of Chinese, Malay and Indian races was approximately 5:4:1.

**Table 2:** Demographic statistics from both study sites and the total of the whole sample.

Demographic variables	Klang Valley (N = 97)	Iskandar Malaysia (N = 117)	χ^2^	df	p-value	Total
	%	%				%
**Gender**			1.355	1	.244	
Male	43.3	51.3				47.7
Female	56.7	48.7				52.3
**Age (years)**			3.951	4	.413	
< 18	1.0	4.3				2.8
18–20	15.5	10.3				12.6
21–55	67.0	69.2				68.2
56–64	15.5	13.7				14.5
> 64	1.0	2.6				1.9
**Ethnicity**			4.845	2	.089	
Malay	44.3	29.9				36.4
Chinese	44.3	57.3				51.4
Indian	11.3	12.8				12.2
**Education level**			13.355	3	.004*	
Primary	9.3	4.3				6.6
Secondary	9.3	28.2				19.6
Tertiary	73.2	59.0				65.4
Postgraduate	8.2	8.5				8.4
**Employment status**			10.704	6	.098	
Full time	26.8	34.2				30.8
Part time	1.0	7.7				4.7
Self-employed	6.2	5.1				5.6
Retired	3.1	5.1				4.2
Housewife	7.2	2.6				4.7
Student	50.5	42.7				46.3
Unemployed	5.2	2.6				3.7
**Employment sector**	N = 36	N = 61	6.174	3	.103	N = 97
Non-environmental government sector	16.7	4.9				9.3
Non-environmental private industry	41.7	63.9				55.7
Government / private educational institution	19.4	13.1				15.5
Government / private environmental sector	22.2	18.0				19.6
**Household monthly income**	(N = 71)	(N = 86)	0.220	4	.994	(N = 157)
< RM2500	26.8	24.4				25.5
RM 2501–5000	26.8	26.7				26.8
RM 5001–7500	14.1	14.0				14.0
RM 7501–10,000	14.1	14.0				14.0
> RM10,000	18.3	20.9				19.7
**Household vehicles no**.	(N = 90)	(N = 113)	11.823	4	.019*	(N = 203)
None	3.3	5.3				4.4
1	22.4	14.2				18.7
2	21.1	41.6				32.5
3	28.7	17.7				21.7
>3	24.4	21.2				22.7
**Fuel type**	(N = 92)	(N = 110)	0.297	2	.862	(N = 202)
Diesel	5.4	4.5				5.0
Petrol	81.5	80				80.7
Both	13.0	15.5				14.4
**Have children**	17.5	27.4	2.900	1	.089	22.9
**Family members have respiratory disease / health condition caused by air pollution**	16.5	6.8	4.967	1	.026*	11.2
**Family members have been hospitalized due to respiratory diseases**	15.5	10.3	1.304	1	.253	12.6

N values under parentheses in the table represents the amount of valid answers in that variable as some respondents chose to not answer certain questions.

* p-value < .05

The populations from KV and IM differed significantly in only two demographic parameters. Respondents from KV tend to have higher education level and were more likely to have more vehicles in the household, as compared to IM’s respondents. Other variables such as employment status, income, age and parenthood did not vary significantly between the two populations.

### Health condition in relation to air pollution

Respondents from IM reported significantly fewer cases of air pollution-caused respiratory disease among the household (6.8% vs 16.5%; *p* < .05; Table 2). IM respondents also reported a smaller percentage of ‘family members being hospitalized due to air pollution’ as compared to KV (10.3% vs 15.5%), though this difference was not statistically significant (p > .05).

### Perception of current air quality

**Fig 1:**
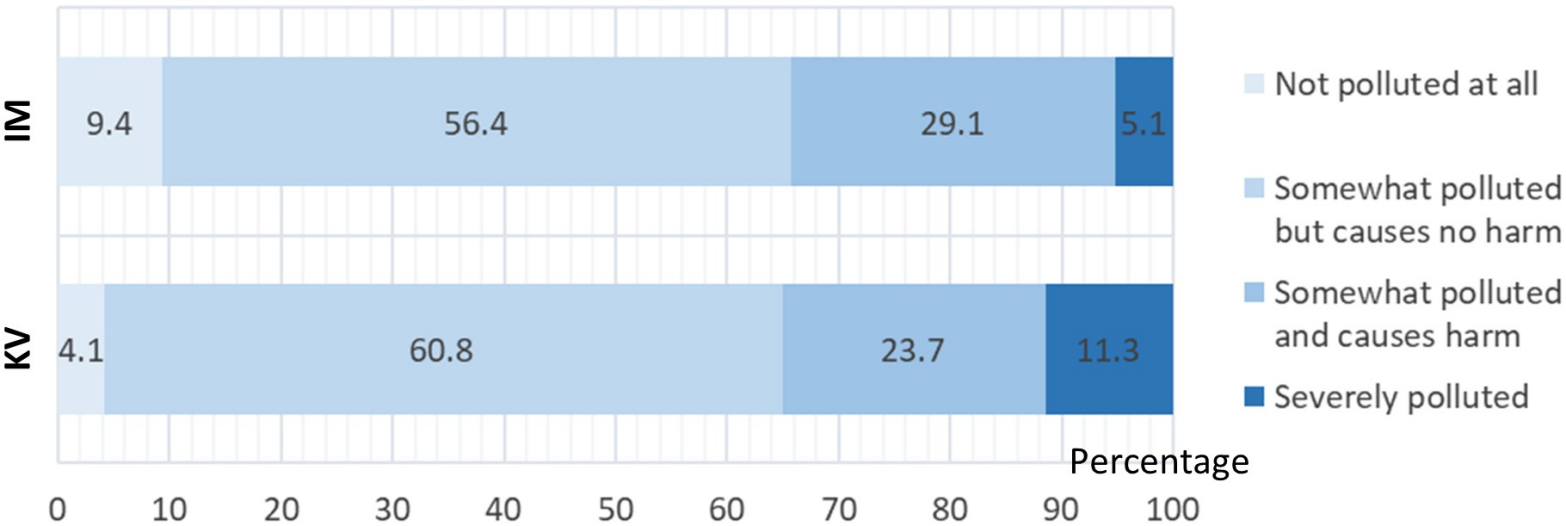
Respondents’ perception on the atmospheric condition in Iskandar Malaysia (IM) and Klang Valley (KV).

Most respondents rated the atmospheric condition at their residential areas as ‘somewhat polluted but causes no harm’, with 66% from IM and 65% from KV being positive towards the air quality (Fig 1). However, there was a slight increase of respondents from KV who perceived their air as ‘severely polluted’ (11%) compared to IM (5%).

**Fig 2:**
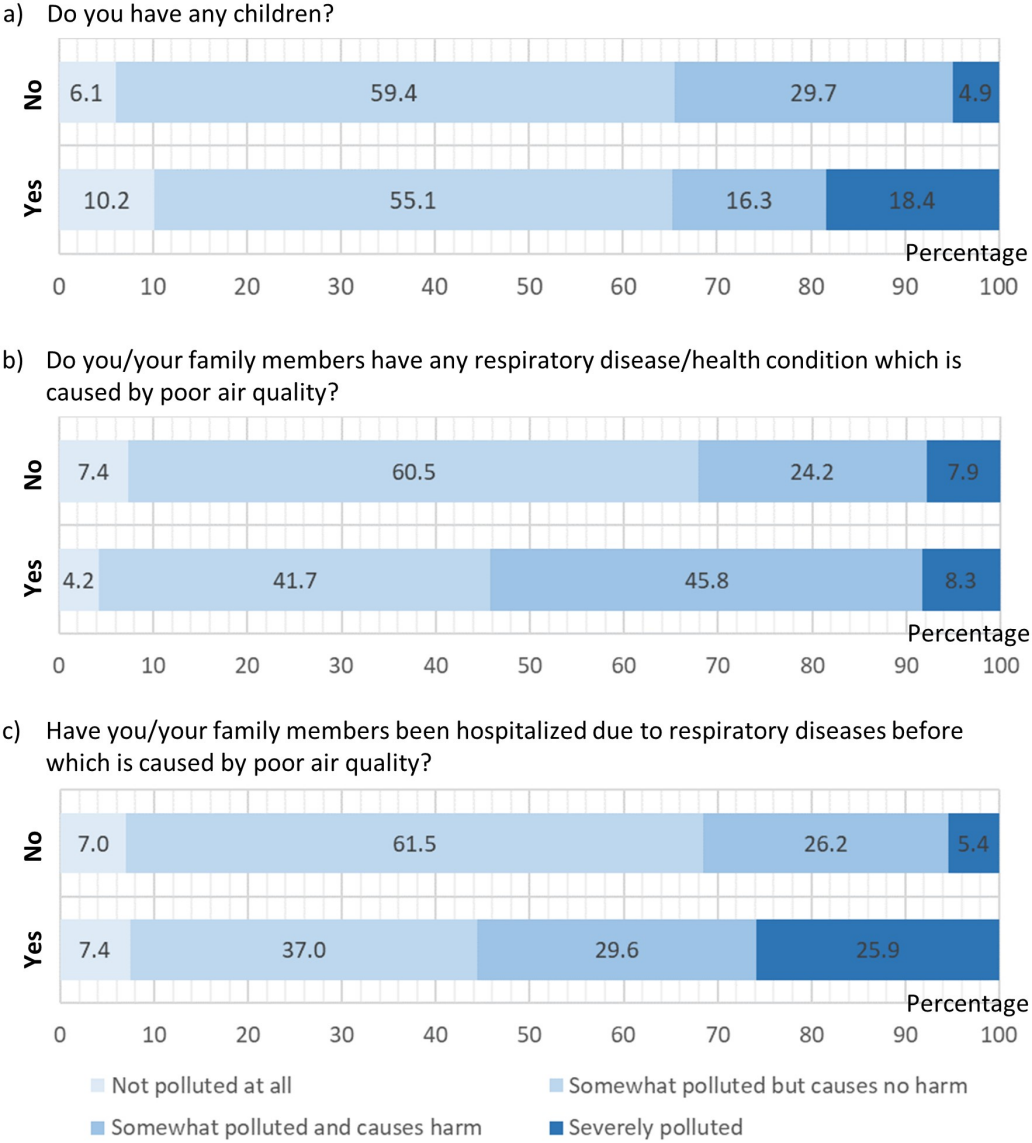
Comparison of respondent’s perception of air quality. Perception levels reported among levels of (a) parenthood, (b) sickness and (c) hospitalization; x-axis indicates percentage.

Vulnerability to air pollution might have influenced perception in respondents. For example, respondents who tend to report the atmospheric condition as ‘severely polluted’ were more likely to have children (χ^2^ = 12.272, df = 3, *p* = .007; Fig 2a). Respondents who stated that they or their family members have had respiratory disease were more likely to view air quality as ‘somewhat polluted and causes harm’ and ‘severely polluted’ (χ^2^ = 5.355, df = 3, *p* = 0.148; Fig 2b), and those being hospitalized showed a larger and statistically significant difference in perception of air quality (χ^2^ = 15.105, df = 3, *p* = 0.002; Fig 2c).

### Perception of air pollution factors

**Fig 3:**
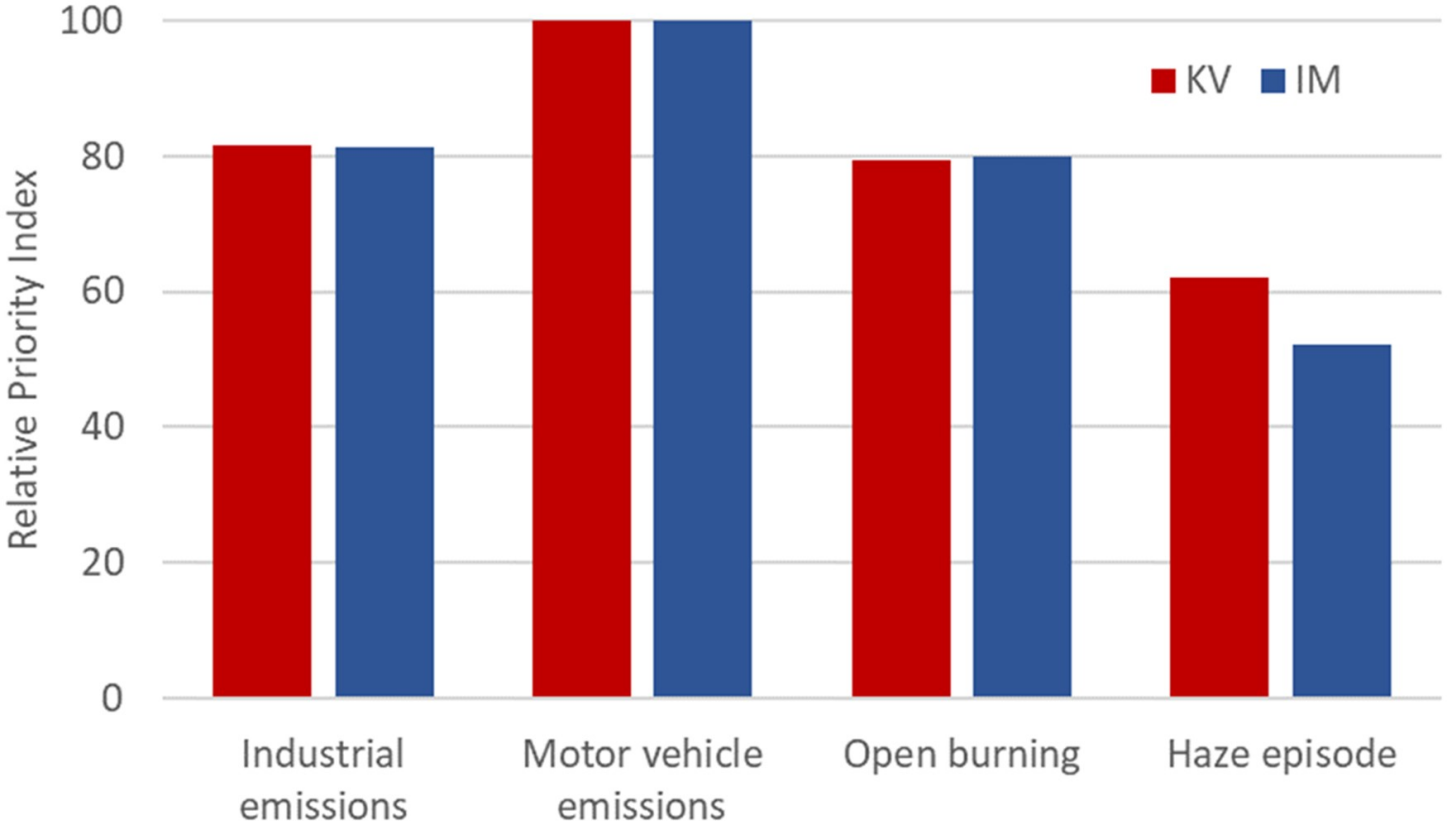
Respondent’s perception of the relative importance of factors contributing to air pollution in Iskandar Malaysia (IM) and Klang Valley (KV).

To obtain a more comprehensive picture of how respondents perceive the air quality, respondents were asked to rank which factor they believed contributes the most to the air pollution at their residential areas. Results showed that respondents in both KV and IM ranked ‘motor vehicle emissions’ as the most significant contributor (RPI = 100), ‘industrial emissions’ as the second highest factor (RPI = 81.67/81.25 in KV and IM respectively), followed closely by ‘open burning’ (RPI = 79.44/79.91), and, with much lower priority, ‘haze episode’ (RPI = 62.22/52.23). Overall, the pattern of the respondents’ choice was not significantly different in the two study sites (χ^2^ = 0.539, df = 212, *p* > .05; Fig 3).

### Awareness of air pollution related information

**Fig 4:**
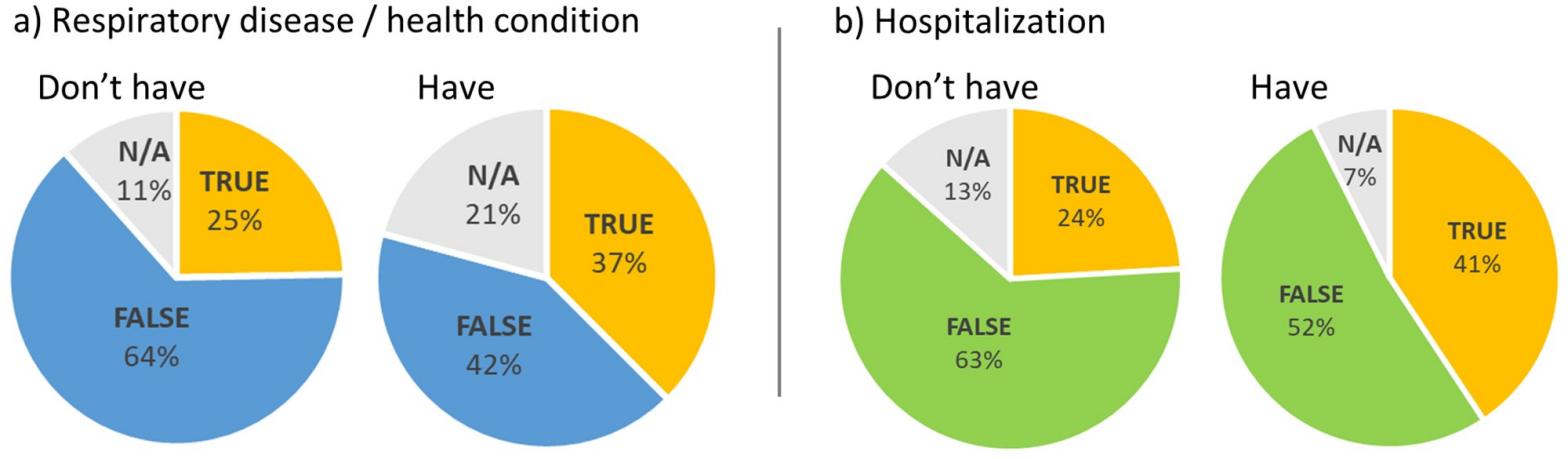
Responses to the statement ‘respiratory diseases were the leading cause of death among Malaysians’. Respondents who “have respiratory disease” or “have been hospitalized” due to air pollution were more likely to think that this false statement is true.

The ‘awareness of air pollution’ was defined by the awareness score obtained from the respondents. The mean (±SD) awareness score of the entire sample was 4.1 ± 1.7 (respondents’ score range = 0 to 9, due to the negative scoring applied for incorrect answers). The awareness scores among different demographic variables was statistically tested and is summarized in Table 3. There was no significant difference among the awareness level of populations from KV and IM (p > .05, Table 3). Female respondents had significantly lower awareness than males (3.8 ± 1.7 vs. 4.3 ± 1.6, *p* < .05). Factors such as age, income, employment sector, education level, income and parenthood had no significant effect on awareness level. Respondents who had respiratory disease or had been hospitalized showed no significant difference in overall awareness level compared to those who did not (p > .05, Table 3). However, when we analysed the sample responses against a false statement “respiratory diseases were the leading cause of death among Malaysians”, we found similar patterns among both aforementioned subsamples, with a higher percentage of them believing respiratory diseases were the primary lethal cause in Malaysia (Fig 4).

**Table 3:** Distribution of awareness among different demographics.

**Demographic variables**	**Knowledge**	
	**Mean ± SD**	
**Overall**	4.1 ± 1.7	Range = 0–9
**Place**		t = 0.872
Klang Valley	4.2 ± 1.5	df = 212
Iskandar Malaysia	4.0 ± 1.8	*p* = .3844
**Gender**		t = 2.210
Male	4.3 ± 1.6	df = 212
Female	3.8 ± 1.7	*p* = .0282*
**Age (years)**		df = (4, 209)
< 18	3.7 ± 2.0	F = 1.990
18–20	3.7 ± 1.7	*p* = .0973
21–55	4.1 ± 1.7	
56–64	4.8 ± 1.3	
> 64	3.5 ± 1.4	
**Ethnicity**		df = (2, 211)
Malay	4.4 ± 1.8	F = 2.286
Chinese	4.0 ± 1.7	*p* = .1042
Indian	3.6 ± 2.1	
**Education level**		df = (3, 210)
Primary	3.9 ± 1.6	F = 0.451
Secondary	3.9 ± 1.8	*p* = .7166
Tertiary	4.2 ± 1.6	
Postgraduate	4.2 ± 1.8	
**Employment sector**		df = (3, 93)
Non-environmental government sector	4.1 ± 1.0	F = 0.081
Non-environmental private industry	4.3 ± 1.8	*p* = .9704
Government / private educational institution	4.2 ± 1.6	
Government / private environmental sector	4.4 ± 1.6	
**Household monthly income**		df = (4, 152)
< RM2500	3.7 ± 1.6	F = 1.616
RM 2501–5000	4.0 ± 1.6	*p* = .173
RM 5001–7500	4.7 ± 1.8	
RM 7501–10,000	4.4 ± 1.9	
> RM10,000	4.2 ± 1.3	
**Household vehicles no**.		df = (4, 198)
No car	4.5 ± 2.0	F = 0.338
1	3.9 ± 1.4	*p* = .8523
2	4.1 ± 1.8	
3	4.0 ± 1.6	
>3	4.2 ± 1.7	
**Fuel type**		df = (2, 199)
Diesel	4.0 ± 1.2	F = 0.404
Petrol	4.1 ± 1.7	*p* = .6682
Both	3.8 ± 1.6	
**Have children**		t = 0.00
No	4.1 ± 1.7	df = 212
Yes	4.1 ± 1.5	*p* > .99
**Family members have respiratory disease / health condition caused by air pollution**		t = 0.560
No	4.1 ± 1.6	df = 212
Yes	3.9 ± 2.0	*p* = .5760
**Family members have been hospitalized due to respiratory diseases**		t = 0.286
No	4.1 ± 1.7	df = 212
Yes	4.0 ± 1.7	*p* = .7754

* p-value < .05

** p-value < .01

### Opinions on environmental protection

**Fig 5:**
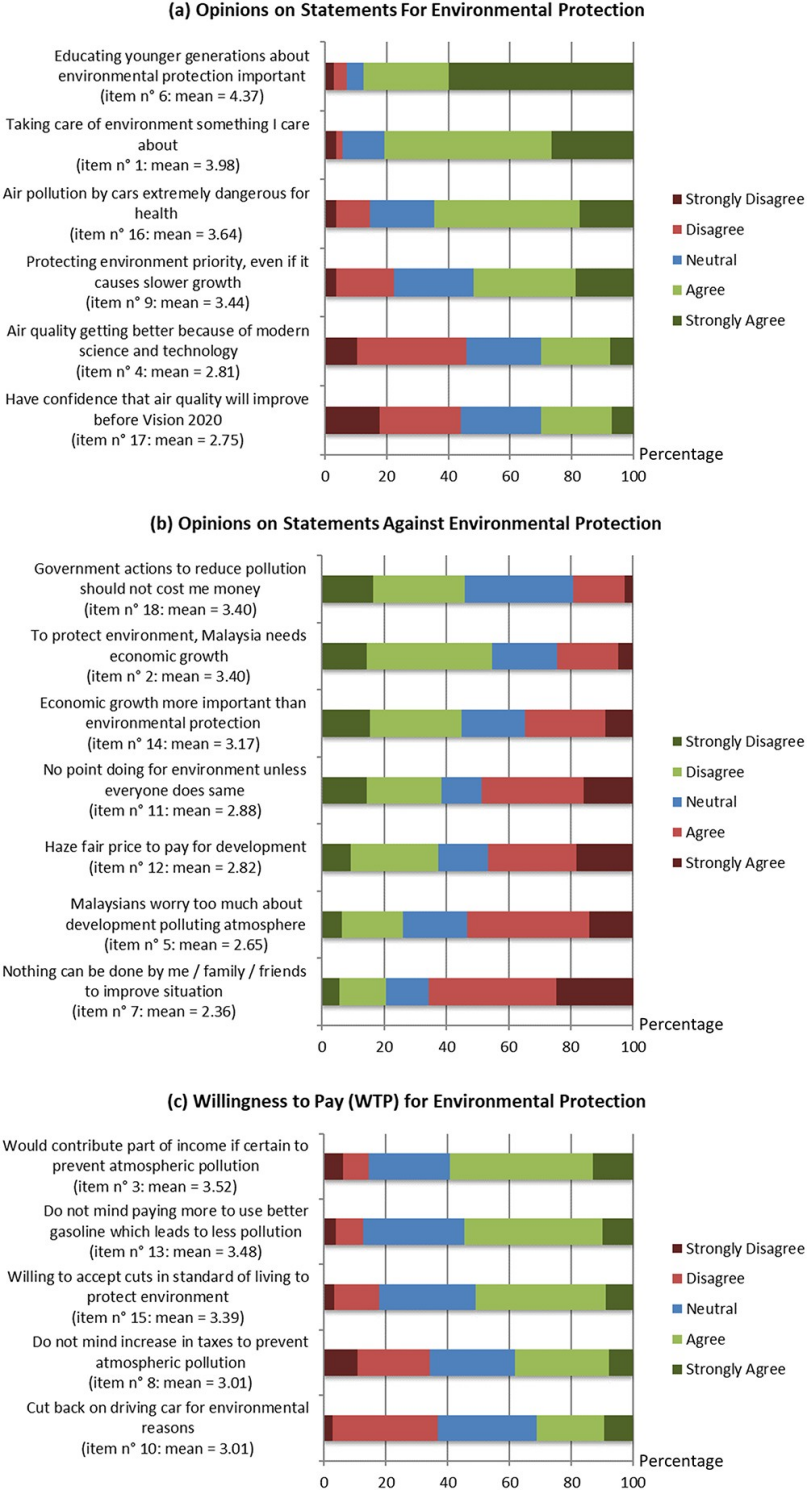
Opinions on statements pro environmental protection (a), statements against environmental protection (b), and statements on Willingness to Pay (WTP) for environmental protection (c). The proportion of participant responses for each question is shown in the chart. The statements against environmental protection (b) were reverse scored, with the strongly disagree response correlating with the highest pro-environmental opinion. Fig 5. Mean pro-environmental scores are shown next to each question on a scale of 1–5. Pro-environmental opinions are indicated by shades of green in all panels.

Attitude statements consisted of a mix of statements that were pro environmental protection and statements that placed environmental protection as unimportant or secondary to something else, such as economic growth (Fig 5a, 5b and 5c). The mean scores for the statements for environmental protection were all greater than 3.0 (neutral), with the strongest agreement observed for general statements, such as “Educating younger generations about the knowledge of environmental protection (e.g. encourage carpool) is important” (mean = 4.37), or “Taking care of the environment is something I really care about” (mean = 3.98). More than 80 percent of respondents agreed or strongly agreed with both of these statements (Fig 5a). Mean scores that were closest to neutral were statements that required individual action on the part of the respondent, such as “I often cut back on driving a car for environmental reasons” (mean = 3.01), or “I do not mind an increase in taxes if the extra money is used to prevent further atmospheric pollution” (mean = 3.01), where less than 40 percent of respondents agreed or strongly agreed with these statements (Fig 5c). Another statement that required individual action had substantially higher agreement from respondents, “I would contribute part of my income if I were certain that the money would be used to prevent atmospheric pollution” (mean = 3.52), with nearly 60 percent of respondents either agreeing or strongly agreeing with it (Fig 5c).

There was broader variation in responses to statements that were against environmental protection, which were reverse scored when calculating mean pro-environmental opinion scores. The lowest agreement and therefore highest pro-environmental scores were seen in responses to questions that downplayed environmental protection, such as “In order to protect the environment, Malaysia needs economic growth” (mean = 3.40) and “Malaysia’s government has to reduce atmospheric pollution, but it should not cost me any money” (mean = 3.40), with less than 25 percent of respondents agreeing or strongly agreeing with either of these statements (Fig 5b). The highest agreement and therefore lowest pro-environmental scores were seen in responses to statements that framed these issues as beyond the scope of an individual’s action, such as “Malaysians worry too much about industrial development polluting the atmosphere and degrading human’s health” (mean = 2.65) and “Nothing can be done by me or my family / friends to improve the current atmospheric situation” (mean = 2.36), with a majority of respondents agreeing or strongly agreeing with these statements (Fig 5b).

### Willingness To Pay for environmental protection

As explained in the *materials and methods* section, we defined the items clustered among the initially intended conative component of a potential attitude scale as ‘willingness-to-pay’ (WTP hereafter) for air pollution prevention control. As mentioned previously, these items had properties that allowed it to be treated as a scale (Fig 5c). Thus, the sample’s levels of WTP were explored as follows. The mean (±SD) WTP score of the entire sample was 3.3 ± 0.6 (range 1–4.8). The WTP level among different demographic variables was summarized in Table 4 and statistically tested. Respondents who owned more than three vehicles in their household showed a significantly higher WTP (4.1 ± 0.3, *p* < .05); respondents who had family members who suffered from respiratory disease had significantly higher WTP compared with those who did not (3.3 ± 0.7 vs 3.0 ± 0.5, *p* < .05). Other demographic factors such as gender, age, education level, income, and parenthood did not have a significant effect on the level of WTP. We also found no significant relationship between WTP and awareness level, nor between WTP and the perception of air quality.

**Table 4:** Levels of Willingness To Pay (WTP) for environmental protection among different demographics.

	Mean ± SD			Mean ± SD	
**Place**		t = 0.000	**Household monthly income**		df = (4, 152)
Klang Valley	3.3 ± 0.7	df = 212	** **< RM2500	3.5 ± 0.7	F = 1.184
Iskandar Malaysia	3.3 ± 0.6	*p* = 1.000	** **RM 2501–5000	3.3 ± 0.7	*p* = 0.3201
**Gender**		t = 0.000	** **RM 5001–7500	3.2 ± 0.6	
Male	3.3 ± 0.7	df = 212	** **RM 7501–10,000	3.2 ± 0.5	
Female	3.3 ± 0.6	*p* = 1.000	** **> RM10,000	3.3 ± 0.6	
**Age (years)**		df = (4, 209)	**Household vehicles no**.		df = (4, 198)
< 18	3.4 ± 0.3	F = 0.918	** **None	3.4 ± 0.6	F = 21.569
18–20	3.5 ± 0.7	*p* = 0.4545	** **1	3.4 ± 0.5	*p* = 0.000
21–55	3.3 ± 0.6		** **2	3.2 ± 0.6	
56–64	3.2 ± 0.8		** **3	3.2 ± 0.7	
> 64	3.1 ± 1.4		** **>3	4.1 ± 0.3	
**Ethnicity**		df = (2, 211)	**Fuel type**		df = (2, 199)
Malay	3.4 ± 0.7	F = 2.079	** **Diesel	3.3 ± 1.0	F = 0.000
Chinese	3.2 ± 0.6	*p* = 0.1276	** **Petrol	3.3 ± 0.6	*p* = 0.999
Indian	3.3 ± 0.8		** **Both	3.3 ± 0.7	
**Education level**		df = (3, 210)	**Have children**		t = 0.945
Primary	3.3 ± 1.0	F = 1.140	** **No	3.3 ± 0.6	df = 212
Secondary	3.4 ± 0.5	*p* = 0.3338	** **Yes	3.2 ± 0.8	*p* = 0.3459
Tertiary	3.2 ± 0.6		**Family members have respiratory disease / health condition caused by air pollution**		t = 2.638
Postgraduate	3.3 ± 0.8				
**Employment sector**		df = (3, 93)	** **No	3.0 ± 0.5	df = 212
Non-environmental government sector	3.0 ± 0.7	F = 1.528 *p* = 0.2126	** **Yes	3.3 ± 0.7	*p* = 0.009
Non-environmental private industry	3.3 ± 0.6		**Family members have been hospitalized due to respiratory diseases**		t = 0.754
Government / private educational institution	3.6 ± 0.9		** **No	3.3 ± 0.6	df = 212
Government / private environmental sector	3.3 ± 0.7		** **Yes	3.4 ± 0.9	*p* = 0.4518

## Discussion

This study examined urban Malaysians’ perceptions of air pollution at their place of residence, their awareness of the causes and impacts of air pollution, their opinions on air pollution, and their willingness to pay for environmental protection. Around two thirds of respondents from both KV and IM were generally positive about air quality where they live, believing the air to be either “not polluted at all” or “somewhat polluted but causes no harm”. This is somewhat surprising because unhealthy air appears to be globally pervasive [46], and in Malaysia, there is particularly strong evidence of harmful levels of pollution in KV (e.g. [10]; [47]; [48]). As such, many Malaysian citizens may not be perceiving a real threat to their health from air pollution. As outlined by Bickerstaff [49], risk perception is influenced by complex social, political and cultural processes, and so further work to understand associated impacts on behaviours related to air pollution would be valuable. Of the minority of respondents who did perceive a harm from air pollution, those in KV had a higher tendency to report the air as severely polluted, perhaps reflecting the reality of higher pollution levels in the more populous KV. Similarly, Awang *et al*. [9] pointed out that particulate pollution has been an issue in KV for decades, with measurements from the early 1980s showing particulate matter to be above standard limits 99% of the time in one measurement site in KV.

We also attempted to determine public perceptions of the factors contributing to the air pollution in KV and IM. The two populations had similar perceptions of ‘motor vehicle emissions’ as the major contributing factor, followed by industrial emissions, and other burning sources such as local burning and forest fires. This perception is consistent with Makmom Abdullah *et al*. [47] and official statistics [12], which suggest that motor vehicles are the major source of airborne pollutants in Malaysia. Nevertheless, despite most respondents (65%) believing that pollution from vehicles endangers health, only a minority (31%) reported often reducing driving for environmental reasons. Bazrbachi *et al*. [31] reported that most Malaysians who rejected the idea of using public transport and would rather drive private vehicles had an unfavourable impression of the accessibility and efficiency of local public transportation. These new findings highlight the potential of improved public transport options in combating urban air pollution in Malaysia.

We found perceptions of air pollution to be influenced by aspects of vulnerability to its health impacts. For example, respondents with children were more likely to perceive air as severely polluted. This may relate to the intuitive parental reaction that recognises the long-term effects of local air pollution, which is more pertinent to children [50] and thus increase perceptions of a health-related problem [7]. In addition, respondents whose family members were suffering from respiratory disease were more likely to perceive conditions as harmful and more likely to believe (incorrectly) that respiratory disease is the leading cause of death among Malaysians. Together, these findings suggest direct experience among Malaysians of health problems perceived to be caused by air pollution is an important determinant of perceptions of air pollution. They are consistent with work (e.g. [49]) emphasizing the centrality of practical everyday experiences in shaping such perceptions.

We found that while male respondents in this study showed a statistically significant higher awareness of air pollution compared to female respondents, the magnitude of the difference was extremely small. The literature in this area is contradictory, with some findings that men are likely to be more scientifically literate and knowledgeable than women [51, 52] and other studies reporting that females tend to have higher awareness towards environmental issues [53, 54]. We found some effect of age on awareness level, but no effect from levels of education and income. Studies such as Qian *et al*. [41] and Rotko *et al*. [55] support this finding, yet others contain opposing results [56, 57]. This lack of clarity in the relationship between awareness level and gender, education, age and income could be due to the different control of demographic background of research subjects and a lack of a uniform assessment criteria for awareness level among different studies. Thus, further research is needed to help understand detailed factors shaping people’s awareness.

We found that respondents with experience of respiratory illness in their family were more willing to pay to protect the atmosphere compared with those without. This result corroborates previous studies, which found that respondents with pollution-related health problems were more eager to improve air quality [31, 58]. It is worth noting that a vast majority of the people surveyed (87%) agreed that educating younger generations about the knowledge of environmental protection is highly important. In relation to this, our results suggest that a relation between awareness of environmental issues (which could be gained through education) and opinions in favour of environmental protection does exist, but is rather weak. This is consistent with previous studies that, applying the theory of planned behaviour [28] to environmental problems, found gaps both in the relation between awareness and attitude [38, 59] and between attitude and behaviour [60, 61, 62], suggesting the investigation of additional variables that could fill the gaps.

As mentioned in the results, while the respondents were largely in agreement with a statement that they deeply cared about the environment (“Taking care of the environment is something I really care about”) and were understanding that actions to combat air pollution would not come without some personal cost (“Malaysia’s government has to reduce atmospheric pollution but it should not cost me any money”), they were also sceptical of actions that could be taken at an individual level to meaningfully combat air pollution (“Nothing can be done by me or my family / friends to improve the current atmospheric situation”). Furthermore, over 50% of the sample also indicated a lack of initiative unless everyone does the same (“There is no point in doing what I can for the environment unless everyone does the same”). This shows that the public has a tendency to feel that their actions have little or no impact as far as reducing air pollution is concerned and this perceived low level of control over environmental problems may discourage affected populations from becoming part of the solution. The role of perceived behavioural control on behavioural intentions and on actual behaviour is well established in the literature [63, 64], and, more specifically, the association of air pollution with perceived ability to deal with the problem has also been demonstrated in several studies (e.g. [65]; [66]).

We also asked questions on the relationship between economic growth and environmental protection. We found a large minority (45%) overtly prioritizing economic growth over environmental protection (“The economic growth of Malaysia is currently more important than environmental protection”) and a larger number of respondents (55%) agreeing or strongly agreeing that “In order to protect the environment, Malaysia needs economic growth”. We also found significant support (52% agreeing or strongly agreeing) for the statement “Protecting the environment should be given priority, even if it causes slower economic growth and some loss of jobs”, and analogously, significant disagreement (47%, similar to [38]) with the statement “Haze is a fair price to pay for economic development”. Overall this is a mixed set of responses, but there appears to be at least some support for the idea that Malaysia’s economic development demands some environmental sacrifices. In this context Malaysia is delicately positioned, with its citizens having experienced rapid development over several decades, but with recent economic instability causing doubts about achieving a long-stated goal of ‘developed nation’ status by 2020 [67]. Further studies will be necessary to assess the importance of changing economic conditions for such attitudes, and also to assess how Malaysian respondents compare with those from other countries (e.g. [34]; [68]).

Despite some respondents’ emphasis on economic growth, many are willing to pay for environmental (here specifically atmospheric) protection. Overall, more than half of the respondents are willing to contribute part of their income (59%), willing to pay more money for cleaner petrol (55%), and willing to accept cuts in their living standard for the sake of environmental protection (51%). These findings are very encouraging for the future success of implementing various environmental policies. However, willingness-to-pay was substantially reduced in the form of tax payment, indicating selective preferences in the nature of payment mechanisms [69, 70]. Among the entire sample, only 38% do not mind the government increasing taxes for the purpose of preventing future air pollution. This lower WTP in the form of a tax may be linked to the implementation of a Goods and Services Tax (GST) in 2015 [71]. The tax implemented by the Malaysian government in 2013 was unpopular and one of the driving forces behind the victory of the opposition Pakatan Harapan coalition, which has now scrapped the tax, in the 2018 Malaysian General Election. Further, it has been shown that citizens’ compliance in paying their tax obligations is greater if there is a perception that their taxes will be used wisely [72] and transparency in government engenders greater trust from citizens. Political events of recent years in Malaysia, such as the 1MDB scandal, may have reduced public faith in government [73], which is reflected in the lower willingness-to-pay towards environmental protection through government taxation. However, if public perception towards the government changes with regards to the newly elected administration, it may be possible that WTP in this context will increase to match WTP in the other contexts.

Despite rigorous design, this survey still has several limitations. For instance, the categorization of survey sample requires further refinement. In this study, participants are only from large urban areas, and the sample is unlikely to be socio-economically representative of the overall population, as nearly 74% of participants had received some tertiary education and 56% worked in private industry. Thus, application of this study’s findings to other groups in Malaysia with different educational backgrounds, professional degrees and other areas should be done with caution, and further country-wide research is required to better understand this wider context. Moreover, open-ended questions need to be incorporated to reveal new problems and deepen the understanding of the respondents’ attitudes towards current air quality. For example, the reason why respondents are not willing to pay more taxes for cleaner air may relate to multiple reasons, such as political views, health conditions, social psychological pressure, and other factors. These need to be addressed more comprehensively in future studies. In the present study, attitudes towards environmental protection have been analysed mainly descriptively because of the lack of internal consistency of the clustered attitude items, which therefore could not form a reliable scale. Further studies should consider the use of new items, specifically developed for and tested in the Malaysian context, in order to develop a Malaysian scale for support of environmental protection.

## Supporting information

S1 File Survey (English). (PDF)

S2 File Survey (Bahasa Melayu). (PDF)

S3 File Raw dataset with responses to Section A (containing personal information on age, ethnicity, gender, income, health conditions etc.) removed to avoid the risk of breaching confidentiality. (XLSX)
