# Supplementary material for: Public awareness and support for environmental protection—A focus on air pollution in peninsular Malaysia
Source: PLoS One. 2019 Mar 14;14(3):e0212206. doi: 10.1371/journal.pone.0212206 (PMC6417846; doi:10.1371/journal.pone.0212206)
Supplement: S1 File — (PDF) [file pone.0212206.s001.pdf]

The University of Nottingham

UNITED KINGDOM · CHINA · MALAYSIA

Study on Public Perception towards Malaysia's Atmospheric Condition

S	E	A	N

Dear Participant,

This is a survey to analyse public awareness and attitudes towards the atmospheric condition in Peninsular Malaysia and the tendency of mass support for environmental protection among Malaysians. The outcome from this survey will be utilized for academic usage in our research project titled "*Public Understanding and Support for Environmental Protection – A Focus on Air Pollution in Peninsular Malaysia*".

In this study, you will be asked to complete this self-completion questionnaire. No special knowledge is required – we are confident that everyone will be able to take part, not just those with strong views or particular viewpoints. Your participation is voluntary and you may discontinue at any time. The following is assured:

- Responses are anonymous, and the individual study results will be confidential. Data will not be traceable to you and it will not be shared with anyone besides the researchers.
- There are no known recognized risks to your participation in this study.

The questionnaire should not take a very long time to complete and we hope you will find it interesting and enjoyable. Thank you for your time and desire to help in this research. If you have any questions, or if you want the study's final report, please contact *Jocelyne Chin* at khby4cyc@nottingham.edu.my.

This survey is initiated by *Chin Yunn Shin Jocelyne*, a final year undergraduate student from BSc(Hons) Environmental Science, School of Environmental and Geographical Sciences, University of Nottingham Malaysia Campus. The whole research project is under the supervision of *Dr. Matthew J. Ashfold*. Shall there be any inquiries, kindly contact Matthew.Ashfold@nottingham.edu.my.

SECTION A – BACKGROUND INFORMATION

How old are you?	What is your gender?	What is your ethnicity?	Where are you currently residing?
☐ Below 18 y/o<br>☐ 18 – 20 y/o<br>☐ 21 – 55 y/o<br>☐ 56 – 64 y/o<br>☐ Above 64 y/o	☐ Female<br>☐ Male	☐ Malay<br>☐ Chinese<br>☐ Indian<br>☐ Others: __________	☐ Klang Valley<br>☐ Johor Bahru<br>☐ Others: __________

What is the highest educational level you have completed?

- ☐ Primary
- ☐ Secondary
- ☐ Tertiary (college / university)
- ☐ Postgraduate (master / PhD)

Do you have any children?

- ☐ Yes
- ☐ No

What is your employment status?

- ☐ Full time
- ☐ Part time
- ☐ Self-employed
- ☐ Retired
- ☐ Housewife
- ☐ Student
- ☐ Unemployed
- ☐ Others: ______________________________

*If your answer above is *Full time* / *Part time* / *Self-employed* / *Retired*, what is (was) your sector of employment?

- ☐ Non-environmental government sector
- ☐ Non-environmental private industry
- ☐ Government / private educational institution
- ☐ Government / private environmental sector

What is your household monthly income (people living together as a family and sharing finances)?

- ☐ < RM 2,500
- ☐ RM 2,501 – 5,000
- ☐ RM 5,001 – 7,500
- ☐ RM 7,501 – 10,000
- ☐ > RM 10,000
- ☐ I prefer not to answer.

How many vehicles in total does your household own?

- ☐ 1
- ☐ 2
- ☐ 3
- ☐ > 3
- ☐ We do not own any car.
- ☐ I prefer not to answer.

*If you own a vehicle, what type of fuel do you normally use?

☐ Diesel ☐ Petrol ☐ Both

Do you / your family members have any respiratory disease / health condition which is caused by poor air quality?

- ☐ No
- ☐ Yes (please specify: ______________________________)

Have you / your family members been hospitalized due to respiratory diseases before which is caused by poor air quality?

☐ Yes ☐ No

SECTION B – AWARENESS ON CURRENT ATMOSPHERIC CONDITION

Please rank the following from 1 to 4 based on what you think contributes the most to the atmospheric pollution at where you live.<br><br>☐ Industrial emissions<br>☐ Motor vehicle emissions<br>☐ Open burning<br>☐ Haze episode<br><br>Please state if you think there are other factors:<br>______________________________<br>______________________________<br>______________________________	Overall, how would you rate the atmospheric condition at where you live?<br>*(Choose one answer only.)*<br><br>☐ Severely polluted<br>☐ Somewhat polluted and causes harm<br>☐ Somewhat polluted but causes no harm<br>☐ Not polluted at all<br><br>Please describe the air quality more in details:<br>______________________________<br>______________________________<br>______________________________

Below are a number of statements. Please tick (✓) whether you believe the statement is more likely to be *TRUE* or *FALSE*. If you *really* have no idea, only then you proceed to choose *N/A*.

The Department of Environment (DOE) of Malaysia measures the country's ambient air quality continuously (24 hours a day).	☐ TRUE ☐ FALSE ☐ N/A
According to the Compendium of Environment Statistics 2015, in year 2014, emission of pollutants to the atmosphere in Malaysia from industrial activity has increased as compared to 2010.	☐ TRUE ☐ FALSE ☐ N/A
Respiratory diseases were the leading cause of death among Malaysians.	☐ TRUE ☐ FALSE ☐ N/A
Vehicles which use diesel fuel emit nitrogen oxides but vehicles using gasoline as a fuel do not.	☐ TRUE ☐ FALSE ☐ N/A
The environmental protection expenditure for air media in Malaysia consists less than 50% of the total expenditure for all media (surface water, noise, air etc.)	☐ TRUE ☐ FALSE ☐ N/A
Severe haze episodes over the past few years were mostly caused by the burning of underground peatland soil.	☐ TRUE ☐ FALSE ☐ N/A
Malaysian Air Pollutant Index (API) standards are more stringent than World Health Organisation (WHO) guidelines.	☐ TRUE ☐ FALSE ☐ N/A
Air quality with API values exceeding 100 are considered likely to cause health effects to the general public.	☐ TRUE ☐ FALSE ☐ N/A
Malaysia is one of the nations which agreed to the ASEAN Agreement on Transboundary Haze Pollution to implement measures to prevent forest fires leading to haze.	☐ TRUE ☐ FALSE ☐ N/A

SECTION C – ATTITUDES TOWARDS ENVIRONMENTAL PROTECTION

Below are statements that describe how you may perceive the attitudes towards environmental protection at personal, public and governmental level. Please use the following scale to indicate your level of agreement or disagreement with each statement. *(Circle your choice)*

1<br>Strongly Disagree	2<br>Disagree	3<br>Neutral	4<br>Agree	5<br>Strongly Agree

1.	Taking care of the environment is something I really care about.	1	2	3	4	5
2.	In order to protect the environment Malaysia needs economic growth.	1	2	3	4	5
3.	I would contribute part of my income if I were certain that the money would be used to prevent atmospheric pollution.	1	2	3	4	5
4.	The air quality in Malaysia is getting better because of modern science and technology.	1	2	3	4	5
5.	Malaysians worry too much about industrial development polluting the atmosphere and degrading human's health.	1	2	3	4	5
6.	Educating younger generations about the knowledge of environmental protection (e.g. encourage carpool) is important.	1	2	3	4	5
7.	Nothing can be done by me or my family / friends to improve the current atmospheric situation.	1	2	3	4	5
8.	I do not mind an increase in taxes if the extra money is used to prevent further atmospheric pollution.	1	2	3	4	5
9.	Protecting the environment should be given priority, even if it causes slower economic growth and some loss of jobs.	1	2	3	4	5
10.	I often cut back on driving a car for environmental reasons.	1	2	3	4	5
11.	There is no point in doing what I can for the environment unless everyone does the same.	1	2	3	4	5
12.	Haze is a fair price to pay for economic development.	1	2	3	4	5
13.	I do not mind paying more money to use better quality gasoline which leads to less pollution.	1	2	3	4	5
14.	The economic growth of Malaysia is currently more important than environmental protection.	1	2	3	4	5
15.	I am willing to accept cuts in my standard of living in order to protect the environment.	1	2	3	4	5
16.	Air pollution caused by cars is extremely dangerous for health.	1	2	3	4	5
17.	I have confidence that the air quality in Malaysia will improve before Wawasan 2020.	1	2	3	4	5
18.	Malaysia government has to reduce atmospheric pollution but it should not cost me any money.	1	2	3	4	5
