# Supplementary material for: Public awareness and support for environmental protection—A focus on air pollution in peninsular Malaysia
Source: PLoS One. 2019 Mar 14;14(3):e0212206. doi: 10.1371/journal.pone.0212206 (PMC6417846; doi:10.1371/journal.pone.0212206)
Supplement: S2 File — (PDF) [file pone.0212206.s002.pdf]

The University of Nottingham

UNITED KINGDOM · CHINA · MALAYSIA

Soal Selidik tentang Persepsi Awam terhadap Keadaan Atmosfera di Malaysia

S	E	A	N

Kepada para responden,

Ini adalah satu kajian untuk menganalisis kesedaran dan sikap terhadap keadaan atmosfera di Semenanjung Malaysia dan kecenderungan sokongan bagi perlindungan alam sekitar dalam kalangan rakyat Malaysia. Hasil daripada kaji selidik ini akan digunakan untuk kegunaan akademik kami dalam projek penyelidikan yang bertajuk "*Public Understanding and Support for Environmental Protection – A Focus on Air Pollution in Peninsular Malaysia*".

Dalam kajian ini, anda akan diminta untuk melengkapkan borang soal selidik ini sendiri tanpa bantuan. Tiada pengetahuan khas diperlukan – kami yakin bahawa semua orang akan dapat mengambil bahagian, bukan sahaja individu yang mempunyai pandangan yang kuat. Penyertaan anda adalah secara sukarela dan anda berhak untuk menarik diri pada bila-bila masa sahaja. Kami akan memastikan:

- Penyertaan anda adalah tertutup dan sebarang keputusan kajian individu akan dirahsiakan. Data tidak akan didedahkan kepada anda dan tidak akan dikongsi bersama sesiapa kecuali penyelidik.
- Tiada sebarang risiko dalam menyertai kajian soal selidik ini.

Soal selidik ini tidak akan mengambil masa panjang untuk melengkapkan dan kami berharap anda seronok menjawab. Terima kasih sudi meluangkan masa untuk membantu kami dalam kajian ini. Jika anda mempunyai sebarang soalan, atau jika anda ingin mengetahui laporan hasil kajian ini, sila hubungi *Jocelyne Chin* di khby4cyc@nottingham.edu.my.

Kajian ini dimulakan oleh *Chin Yunn Shin Jocelyne*, pelajar akhir tahun di *Bsc(Hons) Environmental Science*, *School of Environmental and Geographical Sciences*, University of Nottingham Malaysia Campus. Keseluruhan projek tersebut adalah di bawah penyeliaan *Dr. Matthew J. Ashfold*. Jika anda ada sebarang pertanyaan, sila hubungi Matthew.Ashfold@nottingham.edu.my.

BAHAGIAN A – INFORMASI LATAR BELAKANG

Berapakah umur anda?	Apakah jantina anda?	Apakah bangsa anda?	Di mana tempat tinggal anda sekarang?
☐ 18 tahun ke bawah<br>☐ 18 – 20 tahun<br>☐ 21 – 55 tahun<br>☐ 56 – 64 tahun<br>☐ 64 tahun ke atas	☐ Perempuan<br>☐ Lelaki	☐ Melayu<br>☐ Cina<br>☐ India<br>☐ Lain-lain:__________	☐ Klang Valley<br>☐ Johor Bahru<br>☐ Lain-lain: __________

Apakah tahap pencapaian pendidikan anda yang tertinggi?

- ☐ Sekolah rendah
- ☐ Sekolah menengah
- ☐ Pengajian tinggi (Kolej / Universiti)
- ☐ Siswazah (Sarjana / PhD)

Adakah anda mempunyai anak?

- ☐ Ya
- ☐ Tidak

Apakah status pekerjaan anda?

- ☐ Sepenuh masa
- ☐ Sampingan masa
- ☐ Bekerja sendiri
- ☐ Bersara
- ☐ Suri rumah
- ☐ Pelajar
- ☐ Tidak bekerja
- ☐ Lain-lain: ______________________________

*Jika jawapan anda diatas adalah *Sepenuh masa* / *Sampingan masa* / *Bekerja sendiri* / *Bersara*, apakah jenis sector pekerjaan anda?

- ☐ Sektor kerajaan bukan alam sekitar
- ☐ Industri swasta bukan alam sekitar
- ☐ Institusi pendidikan kerajaan / swasta
- ☐ Sektor alam sekitar kerajaan / swasta

Apakah pendapatan bulanan isi rumah anda (tinggal bersama dan berkongsi kewangan)?

- ☐ < RM 2,500
- ☐ RM 2,501 – 5,000
- ☐ RM 5,001 – 7,500
- ☐ RM 7,501 – 10,000
- ☐ > RM 10,000
- ☐ Saya tidak minat menjawab.

Berapa banyak jumlah kenderaan keseluruhan isi rumah anda menpunyai?

- ☐ 1
- ☐ 2
- ☐ 3
- ☐ > 3
- ☐ Tiada kenderaan.
- ☐ Saya tidak minat menjawab.

*Jika anda memiliki kenderaan, apakah jenis bahan api yang anda biasanya gunakan?

☐ Diesel ☐ Petrol ☐ Kedua-duanya

Adakah anda / ahli keluarga anda mempunyai sebarang penyakit pernafasan / keadaan kesihatan yang disebabkan oleh kualiti udara?

- ☐ Tidak
- ☐ Ya (sila nyatakn: __________________________)

Adakah anda / ahli keluarga anda pernah dimasukkan ke hospital oleh sebab penyakit pernafasan yang disebabkan oleh kualiti udara?

☐ Ya ☐ Tidak

BAHAGIAN B – KESEDARAN TENTANG KEADAAN ATMOSFERA SEMASA

Sila pangkat berikut dari **1** hingga **4** berdasarkan apa yang anda fikirkan menyumbang terbanyak kepada pencemaran udara di tempat tinggal anda.<br><br>☐ Pelepasan perindustrian<br>☐ Pelepasan kenderaan<br>☐ Pembakaran terbuka<br>☐ Episod jerebu<br><br>Sila nyatakan jika anda fikir terdapat faktor lain:<br>____________________<br>____________________<br>____________________	Secara keseluruhan, bagaimana anda menilai keadaan atmosfera di tempat tinggal anda?<br>*(Pilih satu jawapan sahaja.)*<br><br>☐ Tercemar teruk<br>☐ Agak tercemar dan membahaya<br>☐ Agak tercemar tetapi tidak membahaya<br>☐ Tidak tercemar<br><br>Sila jelaskan kualiti udara dengan lebih butir-butir:<br>____________________<br>____________________<br>____________________

Berikut adalah beberapa penyata. Sila tandakan (✓) sama ada anda fikir penyata itu adalah *BENAR* atau *PALSU*. Jika anda tidak mempunyai sebarang pandangan, barulah anda terus untuk memilih *N/A*.

Department of Environment (DOE) Malaysia mengukur kualiti udara negara kita berterusan (24 jam sehari).	☐ BENAR ☐ PALSU ☐ N/A
Menurut *Compendium of Environment Statistics 2015*, pada tahun 2014, pelepasan pencemaran aktiviti industri ke atmosfera di Malaysia telah meningkat berbanding dengan tahun 2010.	☐ BENAR ☐ PALSU ☐ N/A
Penyakit-penyakit pernafasan merupakan punca utama kematian dalam kalangan rakyat Malaysia.	☐ BENAR ☐ PALSU ☐ N/A
Kenderaan yang menggunakan diesel sebagai bahan api akan mengeluarkan *nitrogen oxides* tetapi kenderaan yang menggunakan petrol sebagai bahan api tidak mengeluarkan *nitrogen oxides*.	☐ BENAR ☐ PALSU ☐ N/A
Jumlah perbelanjaan perlindungan alam sekitar bagi media udara di Malaysia terdiri kurang daripada 50% jumlah seluruh perbelanjaan bagi semua media (air, bunyi, udara dan lain-lain).	☐ BENAR ☐ PALSU ☐ N/A
Keadaan jerebu teruk kebelakangan ini disebabkan oleh pembakaran tanah gambut (bahagian bawah tanah, *peatland soil*)?	☐ BENAR ☐ PALSU ☐ N/A
Tahap Standard Indeks Pencemaran Udara (IPU) Malaysia adalah lebih ketat berbanding dengan garis panduan Organisasi Kesihatan Dunia (*World Health Organization, WHO*).	☐ BENAR ☐ PALSU ☐ N/A
Kualiti udara yang melebihi 100 IPU dinilaikan akan menyebabkan kesihatan masalah bagi publik awam.	☐ BENAR ☐ PALSU ☐ N/A
Malaysia adalah salah satu negara yang bersetuju dengan perjanjian ASEAN mengenai jerebu merentasi sempadan untuk melaksanakan langkah langkah bagi mengelakkan kebakaran hutan yang menyebabkan jerebu.	☐ BENAR ☐ PALSU ☐ N/A

BAHAGIAN C – SIKAP TERHADAP PERLINDUNGAN ALAM SEKITAR

Berikut adalah penyata yang menghuraikan tentang perasaan dan pendapat anda terhadap perlindungan alam sekitar di peringkat individu, awam dan kerajaan. Sila gunakan skala tersebut untuk menyatakan sama ada anda setuju ataupun tidak setuju mengenai setiap penyataan. *(Bulatkan pilihan anda.)*

1<br>Sangat tidak setuju	2<br>Tidak bersetuju	3<br>Neutral	4<br>Setuju	5<br>Sangat bersetuju

1.	Menjaga alam sekitar adalah sesuatu yang saya benar-benar prihatin.	1	2	3	4	5
2.	Untuk melindungi alam sekitar, Malaysia memerlukan pembangunan ekonomi.	1	2	3	4	5
3.	Saya akan menyumbang sebahagian pendapatan saya jika saya yakin bahawa wang itu akan digunakan untuk mencegah pencemaran udara.	1	2	3	4	5
4.	Kualiti udara di Malaysia semakin baik kerana sains dan teknologi moden.	1	2	3	4	5
5.	Rakyat Malaysia dah terlalu bimbang tentang pembangunan industri mencemari atmosfera dan mengancam kesihatan manusia.	1	2	3	4	5
6.	Mendidik generasi muda tentang ilmu perlindungan alam sekitar (contohnya menggalakkan perkongsian kereta) adalah penting.	1	2	3	4	5
7.	Tiada apa yang boleh saya / ahli keluarga / kawan saya lakukan untuk memperbaiki keadaan atmosfera semasa.	1	2	3	4	5
8.	Saya tidak kisah tentang kenaikan cukai jika wang tambahan itu digunakan untuk mencegah pencemaran atmosfera.	1	2	3	4	5
9.	Melindungi alam sekitar harus diberi keutamaan, malah jika ia memperlahankan pertumbuhan ekonomi dan menyebabkan kehilangan perkerjaan.	1	2	3	4	5
10.	Saya sering kurangkan memandu kereta atas sebab alam sekitar.	1	2	3	4	5
11.	Tiada gunanya saya membuat apa-apa bagi alam sekitar melainkan jika semua orang bertindak sama.	1	2	3	4	5
12.	Jerebu adalah harga yang layak dibayar terhadap pembangunan ekonomi masa kini.	1	2	3	4	5
13.	Saya tidak kisah membayar lebih banyak wang untuk menggunakan petrol yang lebih baik kualiti yang akan mengurangkan pencemaran.	1	2	3	4	5
14.	Pembangunan ekonomi Malaysia masa kini lebih penting daripada perlindungan alam sekitar.	1	2	3	4	5
15.	Saya bersedia untuk mengurangkan standard hidup saya untuk melindungi alam sekitar.	1	2	3	4	5
16.	Pencemaran udara yang disebabkan oleh kereta amat membahayai kesihatan manusia.	1	2	3	4	5
17.	Saya yakin bahawa kualiti udara di Malaysia akan menjadi lebih baik sebelum Wawasan 2020.	1	2	3	4	5
18.	Kerajaan Malaysia perlu mengurangkan pencemaran udara tetapi ia tidak patut menyebabkan saya bayar sebarang wang.	1	2	3	4	5
